# Plastid genome analysis of three Nemaliophycidae red algal species suggests environmental adaptation for iron limited habitats

**DOI:** 10.1371/journal.pone.0196995

**Published:** 2018-05-08

**Authors:** Chung Hyun Cho, Ji Won Choi, Daryl W. Lam, Kyeong Mi Kim, Hwan Su Yoon

**Affiliations:** 1 Department of Biological Sciences, Sungkyunkwan University, Suwon, Korea; 2 Department of Biological Sciences, University of Alabama, Tuscaloosa, Alabama, United States of America; 3 Marine Biodiversity Institute of Korea, Seocheon, Korea; Texas A&M University College Station, UNITED STATES

## Abstract

The red algal subclass Nemaliophycidae includes both marine and freshwater taxa that contribute to more than half of the freshwater species in Rhodophyta. Given that these taxa inhabit diverse habitats, the Nemaliophycidae is a suitable model for studying environmental adaptation. For this purpose, we characterized plastid genomes of two freshwater species, *Kumanoa americana* (Batrachospermales) and *Thorea hispida* (Thoreales), and one marine species *Palmaria palmata* (Palmariales). Comparative genome analysis identified seven genes (*ycf*34, *ycf*35, *ycf*37, *ycf*46, *ycf*91, *grx*, and *pbs*A) that were different among marine and freshwater species. Among currently available red algal plastid genomes (127), four genes (*pbs*A, *ycf*34, *ycf*35, *ycf*37) were retained in most of the marine species. Among these, the *pbs*A gene, known for encoding heme oxygenase, had two additional copies (*HMOX1* and *HMOX2*) that were newly discovered and were reported from previously red algal nuclear genomes. Each type of heme oxygenase had a different evolutionary history and special modifications (*e*.*g*., plastid targeting signal peptide). Based on this observation, we suggest that the plastid-encoded *pbs*A contributes to the iron controlling system in iron-deprived conditions. Thus, we highlight that this functional requirement may have prevented gene loss during the long evolutionary history of red algal plastid genomes.

## Introduction

The red algal class Florideophyceae comprises 95% (6,748 spp. out of 7,100 spp.) of the Rhodophyta and encompass a biologically diversified group of taxa [1, 2]. Most of the red algal species (>95%) inhabit marine habitats, however about 5% are found in freshwater environments [3]. The Nemaliophycidae, one of five subclasses within Florideophyceae, contains more than half of these freshwater species. This subclass includes both marine and freshwater taxa with three exclusively freshwater orders (Balbianiales, Batrachospermales, Thoreales), six exclusively marine orders (Rhodachlyales, Balliales, Nemaliales, Entwisleiales, Colaconematales, Palmariales), and one order (Acrochaetiales) with both freshwater and marine species [1, 2, 4, 5]. Among the freshwater orders, the Batrachospermales and Thoreales have more than half of the freshwater species in all of the Rhodophyta [6]. Thus, a comparison of Nemaliophycidae plastid genomes of taxa from freshwater and marine habitats may provide insights into environmental adaptation [1].

A previous study demonstrated that the freshwater angiosperm *Najas flexilis* (water nymph) adapted to the aquatic environment from its terrestrial ancestor by the complete loss of the *ndh* gene family in plastid genome [7]. The NDH gene complexes encode for the NAD(P)H dehydrogenase complex that increases photosynthetic efficiency at variable light intensities in terrestrial habitats. In its transition to the aquatic environment, *N*. *flexilis* did not require resistance to high light stress (due to the refractive properties of water) and therefore the *ndh* gene family had been lost. Likewise, similar gene loss or retention events may be present in the evolution of red algal plastid genomes during their transitions from marine habitats to freshwater systems.

To date, 99 florideophycean plastid genomes (cf. 127 red algal plastid genomes including three new genomes) are available in the NCBI organelle database, including 23 Nemaliophycidae that have been detailed in three recent papers [8–10]. To extend our understanding of red algal plastid evolution as it relates to the habitat adaptation, we completely sequenced and annotated three new plastid genomes for Nemaliophycidae, including one marine (*Palmaria palmata*) and two freshwater species (*Kumanoa americana*, *Thorea hispida*). From a comparative analysis of plastid genomes, we seek to identify plastid genes involved in the transition between marine and freshwater red algae and their physiological implications.

## Materials and methods

### Whole genome sequencing and plastid genome construction

Culture strains of two filamentous freshwater species of *Kumanoa americana* (hsy120, isolated by Franklyn D. Ott from a stream in Mississippi, USA) and *Thorea hispida* (hsy077, isolated by F. Ott from the Kaw river in Kansas, USA) were harvested with gentle centrifugations from the culture flask. Thalli of *Palmaria palmata* (commercially sold as dulse) were collected from Reid State Park in Maine, USA on 27 Aug. 2010 by HSY. Genomic DNA was extracted using the DNeasy Plant Mini Kit (Qiagen, Hilden, Germany) and purified by LaboPass™ DNA Isolation Kit (Cosmo Genetech, Seoul, Korea). Genome sequence data were generated using the Ion Torrent PGM (Thermo Fisher Scientific, San Francisco, California, USA) Next-Generation Sequencing (NGS) platform. The sequencing libraries were prepared using the Ion Xpress Plus gDNA Fragment Library Preparation kit for 200 bp or 400 bp libraries. The library amplification and DNA sequencing were conducted by either Ion PGM Template OT2 200 or 400 Kits and Ion PGM Sequencing OT2 200 or 400 Kit for the Ion Torrent PGM platform.

From NGS genome sequencing data, short raw reads (< 50 bp) were removed completely from the analysis and the remainder of raw reads were *de novo* assembled into contigs using CLC Genomics Workbench 5.5.1 (CLC Bio., Aarhus, Denmark) and MIRA3 Assembler [11]. To obtain a plastid consensus sequence, contigs were sorted by tBLASTn (e-value: 1e-10) using the protein sequence of red algal plastid genes as a reference (i.e. *Chondrus crispus*, *Calliarthron tuberculosum*) [12, 13]. After the reassembly process, we obtained the circular plastid genomes and those circular genomes were confirmed by MUMmerplot [14] and by comparing with reference genomes to check for completeness. The sequences were verified with read-mapping tools implemented in CLC Genomics Workbench to correct for any sequencing errors or gaps.

Protein coding genes were manually annotated following the procedure described in Song et al., 2016 [15] using ‘Bacteria and Archaea (11)’ genetic code and the NCBI database for non-redundant (nr) protein sequences. To search the ribosomal RNA sequences, we applied RNAmmer v1.2 Server [16] using the option of Bacteria, and then re-confirmed by BLASTn. Introns, tRNAs, and other small RNA were searched by ARAGORN [17] and Rfam cmscan v1.1 [18]. Several important genes and ambiguous sequences were re-confirmed with PCR amplification followed by Sanger sequencing. The annotated plastid genomes were visualized using OrganellarGenomeDraw v1.2 [19]. Comparative analysis of the genome structure was accomplished using UniMoG v1.0 [20] and Mauve Genome Alignment v2.2.0 [21, 22] through the Geneious plug-in using the default setting [23].

### A statistical test for the habitat-gene correlation

Habitat information of each species was referred from previous studies. Ott [5] summarized detail about the habitats and isolation history for most nemaliophycidaean species. AlgaeBase [2] provided information about authentic references with the type locality and its distribution. Based on the habitat and gene presence/absence information in the plastid genome, we performed a chi-square analysis that assessed the correlation between habitat and putative habitat-specific genes. We used the chi-square test incorporated into R package [24]. The p-value cut-off (<0.01) was adjusted to reject the null hypothesis.

### Phylogenetic analysis

The red algal protein sequences of heme oxygenase were collected from NCBI public databases, which include transcriptome and genome data. The heme oxygenase homologs were obtained by BLASTp against the NCBI database with an adjusted threshold-cut to 500 top matches of red algal heme oxygenase. To discover the nuclear heme oxygenase, we also surveyed heme oxygenase genes from the published [25–28] or unpublished (H.S. Yoon et al.) whole genome data as described in S5 Table. Multiple sequence alignments were performed using MAFFT version 7 [29] with the default options. These alignments were refined manually based on conserved domains. Maximum likelihood-based phylogenetic analysis and bootstrap methods (MLB) were conducted using IQ-TREE [30] with 1,000 ultrafast bootstrap replications. The evolutionary model was ‘LG + I + G4’ [31], which was automatically selected by the model test option incorporated in IQ-TREE. Finally, highly divergent or contaminant sequences, which showed a long-branch or taxonomical mismatch to sister taxa, were removed from the following analysis.

### Protein domain predictions

Protein domains were searched by the NCBI conserved domain searching tool [32] to predict their functions and conserved motifs. To predict gene localization, TargetP [33] and ChloroP [34] were used based on transit peptide sequences. Transmembrane domain regions were identified using TMHMM program [35]. The molecular function of heme oxygenase and its molecular interaction were surveyed based on KEGG pathway [36].

### Gene network analysis

The dataset of heme oxygenase genes for network analysis was collected from NCBI based on BLASTp with an adjusted threshold-cut to the top 500 matches of red algal heme oxygenase (S2 Fig). The genes containing incomplete heme oxygenase domains were removed from the dataset, therefore, only complete domains were used for network analysis [32]. EGN (Evolutionary Gene and Genome Network) was performed to build a gene network based on protein similarity [37]. For comparative analysis, network connection thresholds were set at 1e-05 in e-value, identities at 20%, hits with at least 20% of the shortest sequence, and query coverage of both sequences at 70%. The resulting network was visualized with the aid of Cytoscape program [38].

## Result and discussion

### General features of three plastid genomes

A total of 1.73 Gbp, 1.94 Gbp, and 1.10 Gbp of raw sequence data were produced for *Palmaria palmata*, *Kumanoa americana*, and *Thorea hispida*, respectively (see details in S1 Table). The average coverage for the plastid genomes was 1,003x in *T*. *hispida*, 215x in *K*. *americana*, and 343x in *P*. *palmata*.

**Fig 1:**
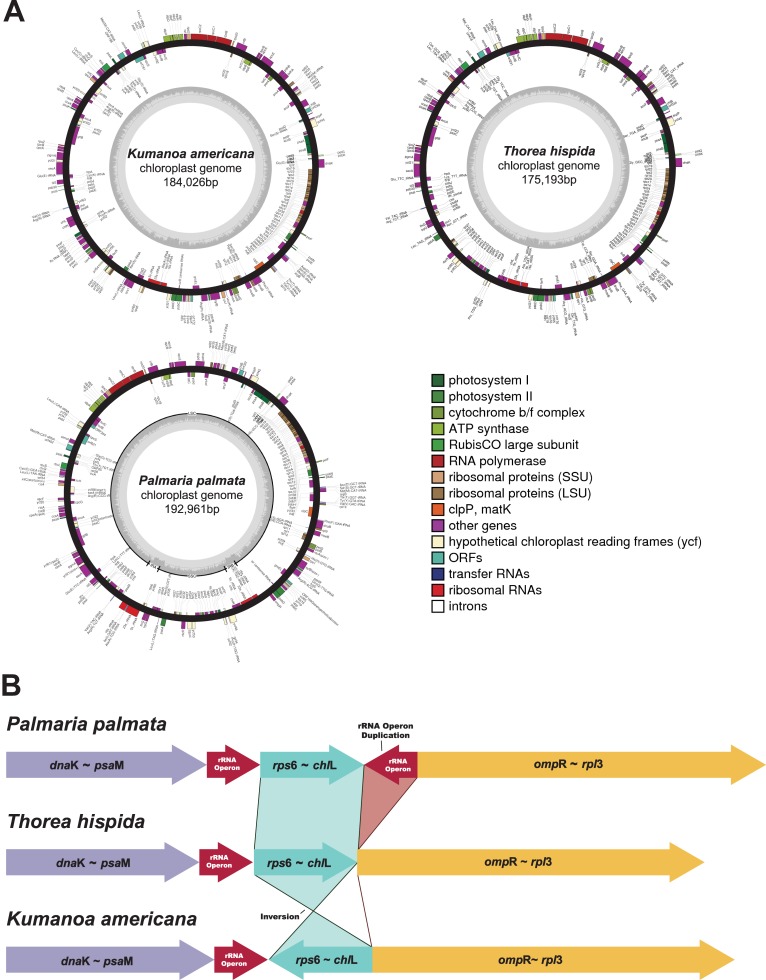
The genome maps of three Nemaliophycidae plastids and their genome structure comparison. (A) Three plastid genome maps of *Kumanoa americana*, *Thorea hispida*, and *Palmaria palmata*. (B) A simplified comparative genome structure between three species based on MAUVE and UniMoG analyses. A large inversion in *rps*6-*chl*L region was observed consistently from two different analyses.

Three complete plastid genomes (Fig 1A) were manually annotated based on published red algal plastid references [39]. Table 1 summarizes the characteristics of plastid genomes of the Florideophyceae [8–10, 12, 13, 39–53]. The total size of the plastid genome was 184,026 bp in *K*. *americana* (GenBank accession number NC_031178), containing 194 protein-coding genes (CDS), while *T*. *hispida* (GenBank accession number NC_031171) was 175,193 bp in size including 192 CDSs. The *P*. *palmata* plastid genome (GenBank accession number NC_031147) was 192,961 bp in size with 203 CDSs. The GC content of *K*. *americana* was 29.3%, which was similar to *T*. *hispida* (28.3%), but lower than that of *P*. *palmata* (33.9%). The high GC content in *P*. *palmata* was more similar to that of the Bangiophyceae (average of 11 spp.: 33.1%) than other Florideophyceae species (average of 102 spp.: 29.3%).

**Table 1:** Comparison of general features for 102 florideophycean plastid genomes.

Subclass	Species	General Characteristics				RNAs		GenBank Accession	Reference
		Total bp	GC %	Introns	CDS	tRNAs	rRNA		
**Hildenbrandio-phycidae**	*Apophlaea sinclairii*	182,437	30.5%	2	190	31	3	NC_031172	[39]
	*Hildenbrandia rivularis*	189,725	32.4%	2	186	31	3	NC_031177	[39]
	*Hildenbrandia rubra*	180,141	31.4%	2	191	31	3	NC_031146	[39]
**Nemaliophycidae**	*Batrachospermum viride-brasiliense*	171,722	28.2%	1	172	30	3	MG252484	[9]
	*Batrachospermum macrosporum*	179,687	28.0%	1	164	37	3	MG252483	[9]
	*Dermonema virens*	184,997	34.1%	2	208	31	3	NC_031655	[8]
	*Dichotomaria marginata*	184,395	28.8%	2	211	31	3	NC_031656	[8]
	*Galaxaura rugosa*	181,215	29.6%	2	207	31	3	NC_031657	[8]
	*Helminthocladia australis*	185,694	32.8%	2	206	31	3	NC_031658	[8]
	*Helminthora furcellata*	184,585	32.1%	2	207	31	3	NC_031654	[8]
	*Hommersandiophycus borowitzkae*	184,728	32.2%	2	205	31	3	NC_031659	[8]
	*Izziella formosana*	183,248	35.0%	2	207	31	3	NC_031660	[8]
	*Kumanoa ambigua*	183,003	28.1%	1	165	28	3	MG252485	[9]
	***Kumanoa americana* hsy120**	**184,025**	**29.3%**	**2**	**201**	**32**	**3**	**NC_031178**	**This study**
	*Kumanoa mahlacensis*	181,361	29.8%	1	166	28	3	MG252486	[9]
	*Liagora brachyclada*	182,937	33.7%	2	207	31	3	NC_031667	[8]
	*Liagora harveyana*	182,933	33.9%	2	208	31	3	NC_031661	[8]
	*Liagoropsis maxima*	189,564	32.1%	2	208	31	3	NC_031662	[8]
	*Nemalion* sp. H.1444	182,930	35.5%	2	204	31	3	LT622871	[8]
	*Neoizziella asiatica*	183,313	33.4%	2	208	31	3	NC_031663	[8]
	***Palmaria palmata***	**192,960**	**33.9%**	**2**	**205**	**34**	**6**	**NC_031147**	**This study**
	*Paralemanea* sp.	180,393	30.5%	1	167	28	3	MG252487	[9]
	*Scinaia undulata*	183,795	35.9%	2	209	31	3	NC_031664	[8]
	*Sheathia arcuata*	187,354	29.9%	2	187	31	6	KY033529	[10]
	*Sirodotia delicatula*	185,555	29.1%	1	164	30	3	MG252489	[9]
	***Thorea hispida* hsy077**	**175,193**	**28.3%**	**2**	**194**	**31**	**3**	**NC_031171**	**This study**
	*Titanophycus setchellii*	183,356	32.7%	2	206	31	3	NC_031665	[8]
	*Trichogloeopsis pedicelleta*	183,497	31.9%	2	206	31	3	NC_031668	[8]
	*Yamadaella caenomyce*	182,460	35.9%	2	206	31	2	NC_031666	[8]
**Corallinophycidae**	*Calliarthron tuberculosum*	178,981	29.2%	2	202	33	3	NC_021075	[12]
	*Sporolithon durum*	191,464	29.3%	2	207	30	3	NC_029857	[44]
**Ahnfeltiophycidae**	*Ahnfeltia plicata*	190,451	32.5%	1	207	31	6	NC_031145	[39]
**Rhodymenio-phycidae**	*Acrosorium ciliolatum*	176,064	25.5%	0	206	33	3	NC_035260	[51]
	*Asparagopsis taxiformis*	177,091	29.4%	2	205	32	3	NC_031148	[39]
	*Bostrychia moritziana*	171,750	28.4%	0	214	34	3	NC_035266	[51]
	*Bostrychia simpliciuscula*	167,514	26.5%	0	202	34	3	NC_035268	[51]
	*Bostrychia tenella*	170,809	28.6%	0	206	34	3	NC_035264	[51]
	*Bryothamnion seaforthii*	175,547	29.5%	0	216	34	3	NC_035276	[51]
	*Caloglossa beccarii*	165,038	26.9%	0	201	33	3	NC_035269	[51]
	*Caloglossa intermedia*	166,397	31.0%	0	209	32	3	NC_035265	[51]
	*Caloglossa monosticha*	165,111	28.2%	0	200	32	3	NC_035263	[51]
	*Ceramium cimbricum*	171,923	27.6%	0	193	28	3	NC_031211	[48]
	*Ceramium japonicum*	171,634	27.8%	1	202	29	3	NC_031174	[39]
	*Chondrus crispus*	180,086	28.7%	1	208	32	3	NC_020795	[13]
	*Choreocolax polysiphoniae*	90,243	20.5%	0	72	0	3	KP308096	[42]
	*Cliftonaea pectinata*	174,482	28.0%	0	214	34	3	NC_035294	[51]
	*Coeloseira compressa*	176,291	29.0%	0	202	30	3	NC_030338	[45]
	*Dasya binghamiae*	177,213	25.6%	0	199	29	3	NC_031161	[40]
	*Dasya naccarioides*	170,970	27.4%	0	201	33	3	NC_035280	[51]
	*Dasyclonium flaccidum*	170,203	28.1%	0	204	34	3	NC_035287	[51]
	*Dictyomenia sonderi*	168,768	28.3%	0	201	34	3	NC_035297	[51]
	*Digenea simplex*	174,848	29.8%	0	207	34	3	NC_035298	[51]
	*Dipterocladia arabiensis*	173,119	26.2%	0	201	34	3	NC_035257	[51]
	*Dipterosiphonia australica*	169,341	28.8%	0	205	33	3	NC_035288	[51]
	*Gelidium elegans*	174,748	30.2%	1	202	30	3	NC_029858	[44]
	*Gelidium vagum*	179,853	29.9%	1	202	30	3	NC_029859	[44]
	*Gracilaria chilensis*	185,637	29.3%	1	204	30	3	NC_029860	[44]
	*Gracilaria firma*	187,001	28.1%	1	219	32	3	NC_033877	[43]
	*Gracilaria salicornia*	179,757	28.8%	0	206	31	3	NC_023785	[53]
	*Gracilaria tenuistipitata*	183,883	29.2%	0	205	29	3	NC_006137	[49]
	*Gracilariopsis chorda*	182,459	27.4%	1	203	30	3	NC_031149	[39]
	*Gracilariopsis lemaneiformis*	183,013	27.4%	2	206	32	3	KP330491	[50]
	*Grateloupia taiwanensis*	191,270	30.6%	0	234	29	3	NC_021618	[52]
	*Gredgaria maugeana*	167,948	27.6%	0	202	34	3	NC_035290	[51]
	*Herposiphonia versicolor*	166,895	28.2%	0	203	34	3	NC_035279	[51]
	*Kuetzingia canaliculata*	178,949	28.1%	0	218	34	3	NC_035293	[51]
	*Laurenciella marilzae*	172,014	29.7%	0	204	34	3	NC_035259	[51]
	*Lophocladia kuetzingii*	175,085	26.9%	0	221	34	3	NC_035292	[51]
	*Mastocarpus papillatus*	184,382	29.1%	0	206	30	3	NC_031167	[41]
	*Melanothamnus harveyi*	164,979	29.8%	0	207	33	3	NC_035281	[51]
	*Membranoptera platyphylla*	176,159	26.4%	0	193	29	3	NC_032041	[46]
	*Membranoptera tenuis*	176,031	26.2%	0	192	29	3	NC_032399	[46]
	*Membranoptera weeksiae*	176,070	26.2%	0	201	29	3	NC_032396	[46]
	*Ophidocladus simpliciusculus*	168,531	28.1%	0	203	34	3	NC_035284	[51]
	*Osmundaria fimbriata*	183,995	28.3%	0	224	34	3	NC_035262	[51]
	*Periphykon beckeri*	168,283	28.4%	0	202	34	3	NC_035261	[51]
	*Platysiphonia delicata*	171,598	29.1%	0	205	33	3	NC_035258	[51]
	*Plocamium cartilagineum*	171,392	27.2%	1	199	29	3	NC_031179	[39]
	*Polysiphonia brodiei*	169,795	29.0%	0	211	34	3	NC_035272	[51]
	*Polysiphonia elongata*	168,290	28.8%	0	206	33	3	NC_035274	[51]
	*Polysiphonia infestans*	165,237	29.4%	0	207	33	3	NC_035277	[51]
	*Polysiphonia schneideri*	163,271	28.1%	0	203	33	3	NC_035296	[51]
	*Polysiphonia scopulorum*	168,001	29.2%	0	206	34	3	NC_035282	[51]
	*Polysiphonia sertularioides*	166,000	29.6%	0	203	33	3	NC_035270	[51]
	*Polysiphonia stricta*	169,061	29.0%	0	201	34	3	NC_035275	[51]
	*Rhodomela confervoides*	175,951	29.0%	0	210	34	3	NC_035271	[51]
	*Rhodymenia pseudopalmata*	194,153	32.0%	1	202	32	6	NC_031144	[39]
	*Riquetophycus* sp.	180,384	28.8%	1	205	30	4	KX284710	[39]
	*Schimmelmannia schousboei*	181,030	28.6%	1	206	30	3	NC_031168	[39]
	*Schizymenia dubyi*	183,959	30.0%	1	206	30	3	NC_031169	[39]
	*Sebdenia flabellata*	192,140	29.2%	2	207	30	3	NC_031170	[39]
	*Sonderella linearis*	169,619	26.0%	0	201	34	3	NC_035289	[51]
	*Spyridia filamentosa*	175,578	29.3%	0	218	33	3	NC_035285	[51]
	*Symphyocladia dendroidea*	171,837	28.4%	0	210	29	0	NC_035267	[51]
	*Taenioma perpusillum*	163,418	27.6%	0	200	32	3	NC_035295	[51]
	*Thaumatella adunca*	169,659	26.5%	0	203	34	3	NC_035291	[51]
	*Thuretia quercifolia*	174,510	25.9%	0	212	34	3	NC_035286	[51]
	*Tolypiocladia glomerulata*	165,623	29.2%	0	206	33	3	NC_035299	[51]
	*Vertebrata australis*	167,318	28.3%	0	199	33	3	NC_035283	[51]
	*Vertebrata isogona*	167,445	28.3%	0	205	33	3	NC_035278	[51]
	*Vertebrata lanosa*	167,158	30.0%	0	193	28	3	KP308097	[42]
	*Vertebrata thuyoides*	168,951	28.6%	0	208	33	3	NC_035273	[51]

Comparing the genome architecture, two major differences were evident among the three Nemaliophycidae species (Fig 1B). First, two copies of ribosomal RNA (rRNA) operon were present in *P*. *palmata*, whereas *K*. *americana* and *T*. *hispida* have only a single rRNA operon (5S, 23S, 16S rRNA) like as most of florideophycean species (Table 1). It has been reported that the plastid genome structures are highly conserved among four florideophycean subclasses (i.e., Nemaliophycidae, Corallinophycidae, Ahnfeltiophycidae, Rhodymeniophycidae) [39]. Second, *K*. *americana* had a large inversion between *chl*L and rRNA operon region that differs from other two species.

### Specific gene loss in freshwater Nemaliophycidae species

Gene contents were generally conserved among the three Nemaliophycidae plastid genomes, however, there were some differences (S1 Fig, S2 Table). For example, the *ycf*91 gene was present only in two freshwater species of *K*. *americana* and *T*. *hispida*, but absent in marine *P*. *palmata*. In addition, six genes (*ycf*34, *ycf*35, *ycf*37, *ycf*46, *grx*, and *pbs*A) were preserved only in the marine *P*. *palmata*, which were absent in the freshwater *K*. *americana* and *T*. *hispida*. Therefore, these seven genes were candidates of habitat-specific plastid gene (i.e., marine vs. freshwater specific).

In order to broaden the investigation of these putative habitat-specific genes, we extended the survey to include all currently available 127 red algal plastid genomes, which include 16 freshwater species, 109 marine species, and two brackish species (S3 Table). From the data in S3 Table, we calculated both the habitat-specific gene concordance rate and the p-value from the chi-square test for seven genes for whether these genes were correlated to the habitat type. Interestingly, we discovered that four of these genes (*pbs*A, *ycf*34, *ycf*35, and *ycf*46) have higher than 80% concordance rate with statistically significant support (p-values = < 0.01). These results suggest that the presence of these four genes is significantly different between marine and freshwater habitats. For example, the *pbs*A gene showed 84.1% of habitat-specific gene concordance rate (p-value = 3.17E-07). Except for four species (*Bangia atropurpurea*, *Sheathia arcuata*, *Paralemanea* sp., *Sirodotia delicatula*), 12 of 16 freshwater species lacked the *pbs*A gene in the plastid genomes. Likewise, the habitat-specific gene concordance rates of *ycf*34, *ycf*35, *ycf*46 and their p-value were 85.6%, 81.8%, 87.9% and 4.83E-10, 2.42E-03, 5.43E-12, respectively.

**Fig 2:**
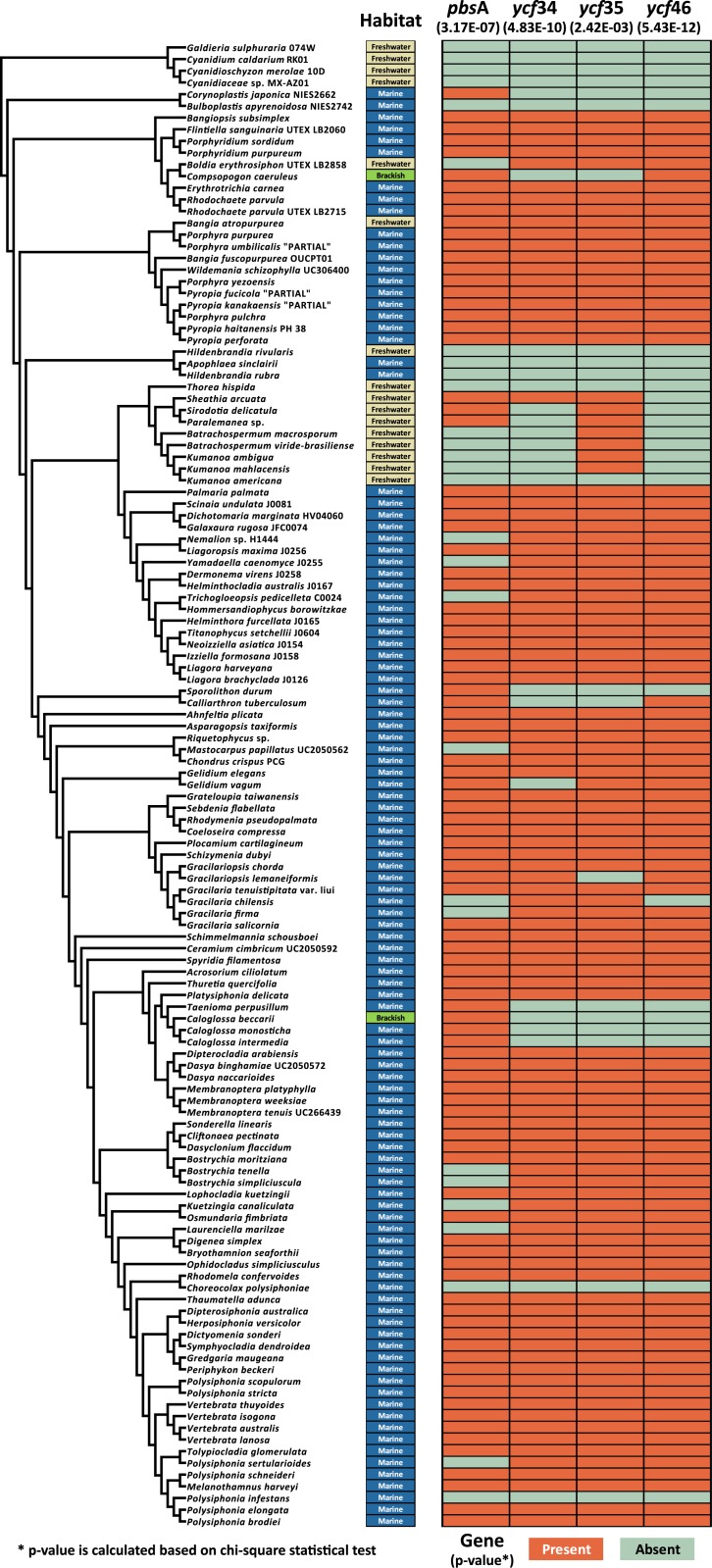
The distribution of four putative habitat-specific genes in the phylogenetic tree. The absence/presence of four genes (*pbs*A, *ycf*34, *ycf*35, *ycf4*6) in 127 species is visualized with habitat information. The maximum likelihood phylogenetic tree was reconstructed based on the concatenated 190 orthologous plastid gene alignment. The dataset used in this analysis is shown in S3 Table.

To inspect the association between the phylogenetic relationship and the habitat-gene pattern, we mapped the presence or absence of these four genes on the ML phylogeny with habitat information (Fig 2). According to the result, the losses (i.e., absence) of four habitat-specific genes were more likely due to a phylogenetic pattern as compared to random events. For instance, all four genes were absent in four Cyanidiales species, which are all freshwater species. Two exclusive freshwater orders of Thoreales (*T*. *hispida*) and Batrachospermales (eight species) were mostly absent of these genes that were clearly different from marine orders that contained these genes (i.e., one species of Palmariales and 16 spp. of Nemaliales). It is interesting that some mangrove species (i.e., *Bulboplastis apyrenoidosa*, *Caloglossa* spp. *Bostrychia* spp.) [54–56] and two parasitic species (i.e., *Polysiphonia infestans*, *Choreocolax polysiphoniae*) [57, 58], which have significantly reduced plastid genomes, lost these genes. However, there were some exceptional cases: two marine species of Hildenbrandiales (*Apophlaea sinclairii*, *Hildenbrandia rubra*) together with one freshwater species (*H*. *rivularis*) were absent of all these genes, same as in two marine Corallinophycidae species (*Sporolithon durum* and *Calliarthron tuberculosum*). Based on this observation (Fig 2), we postulate that four habitat-specific genes (*pbs*A, *ycf*34, *ycf*35, and *ycf*46) were adapted to freshwater habitat during their evolutionary history.

To find a functional relevance of these genes in environmental adaptation, we focused on the *in silico* functional analysis. Because any functions were reported for *ycf* genes, a conserved hypothetical protein family, we selected only on the *pbs*A (heme oxygenase) gene for downstream analyses.

### Heme oxygenase

The function of heme oxygenase is generally known for the degradation of a heme to a biliverdin and is involved in the production of phycobilins [59]. For instance, the heme oxygenase degrades heme that is an essential step in the phycobilin biosynthesis in *Cyanidium caldarium* (Cyanidiophyceae) [60]. During a heme degradation, iron ions are released and those ions play an essential role in the iron recycling pathway [61, 62].

**Fig 3:**
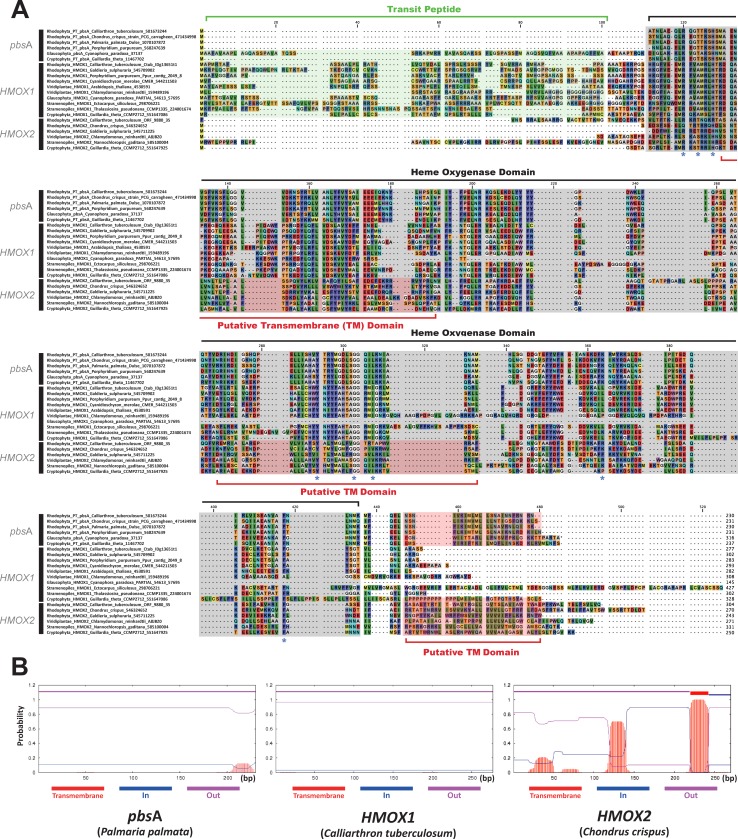
The sequence alignment of heme oxygenase proteins. (A) The alignment of *pbs*A and its homologous proteins. The alignment shows the conserved heme oxygenases amino acid sequences in different lineages. Conserved heme binding pockets are marked as a blue asterisk. N-terminal transit peptides (green) are unique for *HMOX1* proteins, with an exceptional transit peptide of *pbs*A gene in *Cyanophora paradoxa*, which was likely transferred to the nuclear genome independently. Heme oxygenase domain (grey) and putative transmembrane domain (red) are shown. (B) *HMOX2* and *pbs*A contain putative transmembrane domain(s) (TM domain; red box). Multiple TM domains were found in *HMOX2*.

While searching for *pbs*A genes in all available red algal genome data (S5 Table), two additional heme oxygenase genes were newly discovered from nuclear genome data. Both the newly discovered two heme oxygenases and *pbs*A had a conserved heme oxygenase domain (CDD name: HemeO superfamily), with conserved heme binding pockets (blue asterisk in Fig 3). According to recent studies about heme oxygenase in *Chlamydomonas reinhardtii* (Chlorophyta), two distinct types of nuclear-encoded heme oxygenase have been called as *HMOX1* (plant type) and *HMOX2* (animal type) [63]. However, there was no plastidal heme oxygenase (*pbs*A) in green algae and we could not find it from a currently available nuclear genome. Compared to the green algal heme oxygenase, we named three red algal heme oxygenase genes as *HMOX1* and *HMOX2* for nuclear copies and *pbs*A for the plastidal copy.

**Fig 4:**
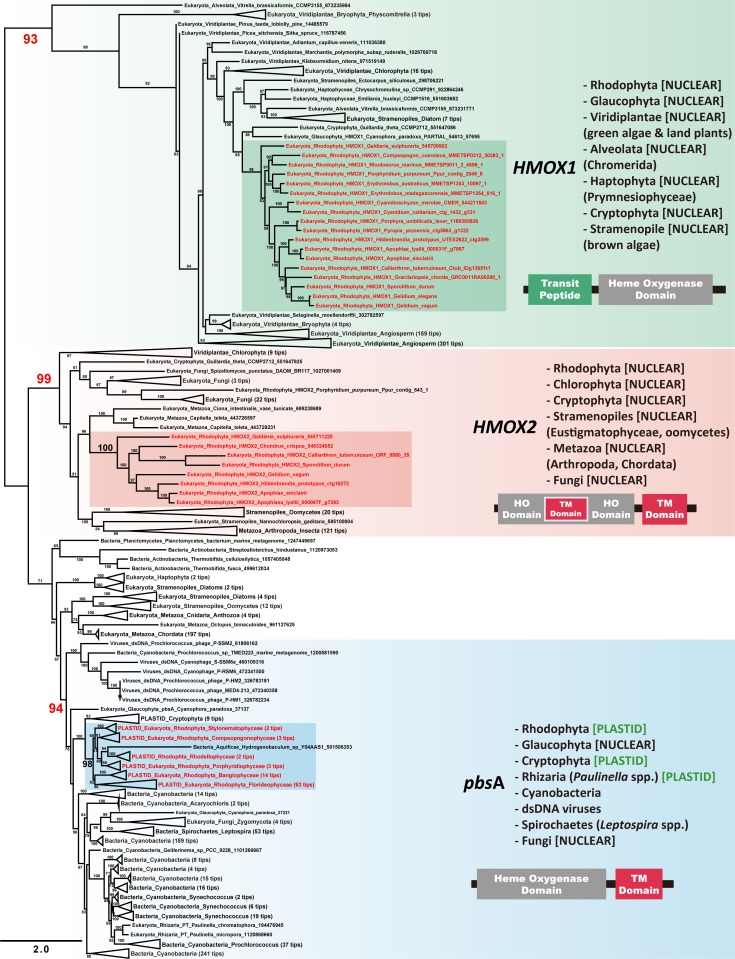
Maximum likelihood tree and schematic diagrams of heme oxygenase proteins. Maximum likelihood (ML) tree based on an alignment of 510 heme oxygenase amino acid sequences from 1,678 taxa. Red algal heme oxygenases had three isotypes of heme oxygenase; *HMOX1*, *HMOX2*, and *pbs*A. *HMOX1* and *HMOX2* were located in the nuclear genome whereas the *pbs*A was encoded in the plastid genome in red algae.

To identify the evolutionary history of three heme oxygenase isotypes, homologs of heme oxygenase were collected from the NCBI database (see details in Materials and Methods). These three distinct types of red algal heme oxygenase were grouped in three clades in the phylogenetic tree (Fig 4).

#### *pbs*A gene

The plastid encoded *pbs*A gene was present in most marine red algal species. Red algal *pbs*A genes were grouped in a highly supported clade with diverse cyanobacteria (94% MLB) (Fig 4), suggesting a cyanobacterial origin. Additionally, *pbs*A was present in the red algal derived plastids of cryptophytes. The *pbs*A homolog was also found in the nuclear genome of the *Cyanophora paradoxa* (Glaucophyta) with an additional extension of the N-terminal transit peptide (e-value = 1e-61; compare to *pbs*A gene of *Porphyridium purpureum*). The alignment for *pbs*A and its homologous proteins were identified to have a functional domain of heme oxygenase (see Fig 3A). Nonetheless, each type of heme oxygenase proteins contained a few differences, such as putative transmembrane domain (red box) in C-terminal extension or targeting domain (green box) in N-terminal extension (Fig 3A). For *pbs*A protein sequences, a transmembrane region was predicted as shown in Fig 3B, where most of the red algal *pbs*A proteins had putative transmembrane domains in the C-terminal. This C-terminal transmembrane domain was also present in *HMOX2* genes, but *HMOX2* genes contained additional putative transmembrane domains inside the functional heme oxygenase domain. None of *HMOX1* genes in red algae were predicted to have a transmembrane domain region in their protein sequences.

One noteworthy discovery was that the *pbs*A gene is generally absent in freshwater species (75%; 12 of 16 spp.), but present in most of the marine red algal species (86%; 100 of 116) (see S3 Table). Heme oxygenase is well known for acting as an iron-controlling factor [61, 64]. It has been demonstrated that transcription of the *pbs*A gene in a unicellular red alga, *Rhodella violacea* (Rhodellophyceae), was up-regulated in iron deprivation conditions [65]. Given these observations, it is highly likely that the *pbs*A gene in red algae assists to uptake of iron in iron deprived marine environment. On the other hand, most freshwater red algae have lost the *pbs*A gene, likely due to this gene being unnecessary or redundant in freshwaters that are typically not as iron limited as marine environments (iron composition in freshwater is ~1,400 times higher than seawater [66]). Although gene content in red algal plastid genomes was highly conserved [39], *pbs*A gene may be a good example for the genomic response to environmental adaption.

#### *HMOX1* gene

Although the nuclear heme oxygenase gene, *HMOX1*, contained a conserved heme oxygenase domain and heme binding motif, it was clearly different from the other heme oxygenase genes in the phylogenetic and gene network analyses. The ML tree (Fig 4) showed that orthologs of *HMOX1* gene form a strongly supported monophyletic group (93% MLB) with relatively long-branches. In addition, the gene network analysis (S2 Fig) also indicated *HMOX1* genes to be clearly separated from the groups of *HMOX2* or *pbs*A despite the conservation of heme oxygenase domain. Unlike *HMOX2* and *pbs*A, protein sequences of the *HMOX1* gene displayed a low similarity (20–30%) to the cyanobacterial heme oxygenase. Interestingly, the *HMOX1* gene had unique N-terminal peptides, which were predicted (via ChloroP software) to target the plastid (Fig 3). N-terminal peptides were present in the photosynthetic eukaryote lineages including the primary endosymbiotic lineages (red algae, Viridiplantae, and a glaucophyte alga *Cyanophora paradoxa*) and red algal-derived secondary plastid groups (i.e., haptophytes, cryptophytes, stramenopiles). While the transit peptides were absent in *HMOX2* and *pbs*A, *pbs*A in *C*. *paradoxa* (Glaucophyta) was located in the nucleus and possesses the additional N-terminal transit peptide like those of *HMOX1* genes. In Chlorophyta species, they retained both *HMOX1* and *HMOX2* in the nucleus, but only the *HMOX1* was found in the streptophytes (charophytes and land plants) [67, 68]. Streptophyta species had several copies of the heme oxygenase gene (*HMOX1*) in their nuclear genome and those genes formed a monophyletic group with other *HMOX1* in the phylogenetic tree (Fig 4). For instance, *Arabidopsis thaliana* contained the biochemically well-characterized heme oxygenase *HY1* with three additional putative heme oxygenase copies (*HO2*, *HO3* and *HO4*) [69]. Analysis of the major biochemical parameters (i.e., enzyme activity depends on pH, temperature, conversion time of heme to biliverdin) demonstrated that activities of these three heme oxygenases (*HO2*, *HO3* and *HO4*) do not different from that of *HY1* [70]. Therefore, unlike other lineages, the four isotypes of *HMOX1* (*HY1*, *HO2*, *HO3* and *HO4*) in land plants likely plays an important role in synthesizing phycobilin, and these four isotypes likely originated from gene duplication events from an ancestral land plant *HMOX1* gene [71, 72].

Because of their low similarities (protein identities: 22.41~27.68%: 1e-05~1e-10) to other homologous proteins, including the cyanobacterial heme oxygenase, the origin of *HMOX1* were still ambiguous. However, *HMOX1* was only present in plastid-bearing eukaryotes and this was the only gene that possesses the plastid-targeting transit peptide among the heme oxygenase families. Therefore, we concluded that *HMOX1* was involved in plastidal function. The phylogenetic position of *HMOX1* in red algal-derived secondary endosymbionts suggested that *HMOX1* was likely derived from secondary endosymbiosis events followed by the gene transfer to the nuclear genome of the host (Fig 4). Indeed, a trafficking experiment in *Chlamydomonas reinhardtii* showed that *Chlamydomonas* nuclear-encoded *HMOX1* gene targets to the plastid [63]. Although none of these studies showed the trafficking of red algal *HMOX1*, it is highly likely that red algal *HMOX1* targets the plastid because of the monophyly of red algae with the Viridiplantae. In Viridiplantae, *Chlamydomonas reinhardtii*, *Arabidopsis thaliana*, and *Ceratodon purpureus* provided experimental evidence for plastid trafficking with the N-terminal extension *HMOX1* [70, 73, 74]. Nevertheless, function of *HMOX1* genes in red algae needs further experimental investigation to elucidate its metabolic pathway.

#### *HMOX2* gene

The red algal *HMOX2*, another nuclear encoded heme oxygenase gene, had one or more putative transmembrane domains in C-terminus (Fig 3). Transmembrane domain structures of red algae were homologous to those of metazoan (e.g., mammalian, amphibian) heme oxygenase [63, 75]. Within the *HMOX2* clade in the phylogenetic trees (Fig 4), red algae were grouped with diverse eukaryotes including chlorophytes, cryptophytes, and stramenopiles as well as non-photosynthetic fungi and Metazoa. Therefore, we would suggest that *HMOX2* was derived from an ancient eukaryotic common ancestor. Gene network analysis and the sequence conservation between the *HMOX2* and *pbs*A in protein alignment supported that the *HMOX2* is related to the iron uptake function. Indeed, it has been reported that *C*. *reinhardtii* captured extracellular heme as an iron source with an association between the *HMOX2* protein and the cytosolic membrane [63].

## Conclusion

Three Nemaliophycidae plastid genomes were completely sequenced and annotated. These included two exclusively freshwater species *Kumanoa americana* and *Thorea hispida* and the marine species *Palmaria palmata*. Until recently, plastid genome data were used mainly for phylogenomic analysis (e.g., [47]), divergence time estimation (e.g., [10]), comparative structural analysis (e.g., [39]), and the development of red algal molecular markers for DNA barcoding studies (e.g., [12]). In this study, we focused on finding genomic clues for an environmental adaptation between marine and freshwater red algal species.

Based on the environment-specific genes of the heme oxygenase family in red algae, we postulate that red algae have adapted efficiently in differing iron concentration conditions through the retention or loss of the heme oxygenase genes. Although this study included only a few freshwater red algal species and was not able to present the direct evidence of correlation between habitat and gene retention, we demonstrated the general trend of red algal plastid gene loss (*ycf*34, *ycf*35, *ycf*46, and *pbs*A) in iron-limited marine habitats.

We also demonstrated different evolutionary strategies of three types of heme oxygenase genes in different lineages (e.g., presence of *HMOX1* with gene duplications [*HY1*, *HO2*, *HO3* and *HO4*), but the absence of *HMOX2* in the streptophytes, which include charophytes and land plants) and habitat conditions (e.g., *pbs*A genes in the marine and freshwater red algal species). It is generally known that plastid genes are in the process of reduction (either complete loss or gene transfer to the host nucleus) after its endosymbiotic origin [76]. However, gene loss from the plastid genome appears functionally constrained as demonstrated in *pbs*A and its gene homologs. Through selective gene retention, red algae successfully adapted to different aquatic environments over billions of years of evolutionary history.

## Supporting information

S1 Fig Venn diagram visualization of comparing gene contents within the three Nemaliophycidae genomes. Six unique genes (*pbs*A, *grx*, *ycf*35, *ycf*36, *ycf*37, *ycf*46) were only found in marine *Palmaria palmata*. For freshwater species, there is only one gene (*ycf*91) that found in *Thorea hispida* and *Kumanoa americana*, but not in *Palmaria palmata*.
(EPS)

S2 Fig Gene network for heme oxygenase. The gene network was constructed by EGN with heme oxygenase database from the public database. We performed analysis with 1e-05 of e-value, 20% of protein identities and 70% of query coverage. The result shows that *HMOX1* are clearly separated from other red algal heme oxygenase.
(EPS)

S1 Table The sequencing information for the plastid genome of three Nemaliophycidae species. (XLSX)

S2 Table A list of genes in the plastid genomes of *Palmaria palmata*, *Kumanoa americana*, and *Thorea hispida*. (XLSX)

S3 Table The habitat-specific gene survey in 127 plastid genomes of red algae. 1) Marine unique gene (only in *Palmaria palmata*: *pbs*A, *grx*, *ycf*35, *ycf*36, *ycf*37, *ycf*46); 2) Freshwater unique gene (not in *Palmaria palmata* but in *Thorea hispida* and *Kumanoa americana*): *ycf*91.
(XLSX)

S4 Table The input data for chi-square statistical test. (XLSX)

S5 Table The protein sequences of heme oxygenase. (XLSX)
